# Exploring prognostic values of DNA ploidy, stroma-tumor fraction and nucleotyping in stage II colon cancer patients

**DOI:** 10.1007/s12672-024-01087-w

**Published:** 2024-06-14

**Authors:** Yutong Lou, Lujing Yang, Shaojun Xu, Luxin Tan, Yanhua Bai, Lin Wang, Tingting Sun, Lixin Zhou, Li Feng, Shenyi Lian, Aiwen Wu, Zhongwu Li

**Affiliations:** 1 Key Laboratory of Carcinogenesis and Translational Research (Ministry of Education), Department of Pathology, Peking University Cancer Hospital & Institute, No.52 Fucheng Road, Haidian District, Beijing, China; 2 Department of Pathology, Beijing Friendship Hospital, Capital Medical University, Beijing, China; 3 Key Laboratory of Carcinogenesis and Translational Research (Ministry of Education), Department of Colorectal Surgery, Peking University Cancer Hospital & Institute, Beijing, China; 4 Gastrointestinal Cancer Center, Peking University Cancer Hospital Inner Mongolian Campus, Affiliated Cancer Hospital of Inner Mongolia Medical University, Hohhot, Inner Mongolia China; 5 Department of Pathology, Peking University Cancer Hospital Inner Mongolian Campus, Affiliated Cancer Hospital of Inner Mongolia Medical University, Hohhot, Inner Mongolia China

**Keywords:** Colon cancer, Ploidy, Stroma, Nucleotyping, Prognosis

## Abstract

**Purpose:**

To assess the prognostic value of three novel biomarkers, DNA ploidy, stroma-tumor fraction, and nucleotyping, seeking for more accurate stratification in stage II colon cancer.

**Methods:**

A total of 417 patients with complete follow up information were enrolled in this study and divided into three clinical risk groups. IHC was performed to examine MSI status. DNA ploidy, stroma and nucleotyping were estimated using automated digital imaging system. Kaplan–Meier survival curves, Cox proportional hazards regression models, and correlation analyses were carried out to process our data.

**Results:**

In the whole cohort of stage II colon cancer, nucleotyping and DNA ploidy were significant prognostic factors on OS in univariate analyses. The combination of nucleotyping and DNA ploidy signified superior OS and DFS. Difference was not significant between low-stroma and high-stroma patients. In multivariable analyses, nucleotyping and the combination of nucleotyping and DNA ploidy were proven the dominant contributory factors for OS. In the low-risk group, we found the combination of nucleotyping and DNA ploidy as the independent prognostic factor statistically significant in both univariate and multivariable, while in the high-risk group, the nucleotyping.

**Conclusions:**

Our study has proven nucleotyping and the combination of DNA ploidy and nucleotyping as independent prognostic indicators, thus expanding the application of nucleotyping as a predictor from high risk stage II colon cancer to whole risks.

**Supplementary Information:**

The online version contains supplementary material available at 10.1007/s12672-024-01087-w.

## Introduction

Colorectal cancer (CRC) has been one of the most common malignant tumors globally as well as in China with its high incidence and mortality. According to an analysis using GLOBOCAN 2020 database, CRC, replacing stomach cancer, has rose up to the second place of new cases with an estimated number of 0.56 million in 2020 in China [1]. The need for postoperative adjuvant chemotherapy remains controversial, especially for stage II patients. Considering the toxicity, costs, inconvenience of treatment, as well as the fact that adjuvant chemotherapy in stage II patients does not always improve the survival, conventional utilization is not recommended [2, 3]. It’s imperative and significant to find implement biomarkers beyond previous clinical grouping criteria [4, 5] to stratify patients more accurately so as to provide basis for guiding clinical treatment and formulating follow-up strategy.

DNA aneuploidy, which is a state of abnormal chromosome number, is a result of an underlying chromosomal instability (CIN). By defects in mitotic checkpoint signaling, cohesion defects, merotelic attachment and multipolar mitotic divisions, there is a chance that chromosome is not partitioned evenly into two daughter cells. For more than a century, DNA aneuploidy has been a common characteristic of tumor cells and proposed to drive tumor progression [6]. Used as a surrogate marker for CIN, aneuploidy is correlated with reduced patient survival and inferior outcome in many cancer types including CRC, and may enhance the tumors’ ability to spread to distant sites, representing an increased possibility of CRC metastasis [7–10].

The tumor cells live in a rich microenvironment consisting of resident fibroblasts, endothelial cells, capillary pericytes, leukocytes and extra-cellular matrix, which is also known as tumor stroma [11]. In normal tissue, stroma acts as a protective area. While in tumor tissue, the tumor-activated stroma may generate dynamic signal transduction pathways and promote the occurrence and development of cancer through continuous paracrine secretion [12].Previous studies have proven that a high stroma ratio is associated with poorer survival in colon cancers [13, 14] as well as in other solid epithelial cancers [15–17].

Genetic alterations, ranging from single nucleotide changes, gene amplifications, to chromosome translocatioins and chromosome number changes, occur in many cancer types. Chromatin reorganization, acting as a main contributor to the high mutation rates in cancer genomes [18], has been studied using nuclear texture analysis, giving information about the spatial arrangement of the pixel gray levels in a digitized microscopic nuclear image [19]. Based on this theory, Kleppe and colleagues developed an automated method to identify aberrant chromatin named nucleotyping, which was demonstrated to be a powerful pan-cancer prognostic tool and can stratify colorectal cancer patients more precisely than microsatellite instability (MSI) [20].

The biomarkers of DNA ploidy, stroma-tumor fraction, as well as nucleotyping, have been clinically validated as prognostic predictors in colorectal cancer within a European population [20, 21]. In recent domestic studies, nucleotyping was confirmed as an independent prognostic factor in high-risk stage II colon cancer [22] and the combination of DNA ploidy, stroma and nucleotyping may be a hopeful biomarker to predict survival and guide chemotherapy decisions [23]. In our study, we sought to explore the standalone and combined predictive functions of the three parameters in a cohort of stage II colon cancer, which was then divided into three risk groups according to ESMO (European Society for Medical Oncology) guidelines [5]. In each group, we assessed the prognostic values and tried to discuss the appropriate population for postoperative chemotherapy.

## Patients and methods

### Patient management

We retrospectively collected specimens from patients of stage II colon cancer according to the 7th edition AJCC TNM Classification criteria who underwent surgical resection between 2009 and 2016 at Peking University Cancer Hospital. The basic information (including age and gender) and clinical information (including whether or not suffering intestinal obstruction or perforation and whether or not receiving postoperative chemotherapy) were completely recorded. None of the patients had undergone neoadjuvant chemoradiotherapy before surgery. We used formalin-fixed and paraffin-embedded (FFPE) samples, which were cut into sections using hematoxylin–eosin (H&E) staining. The pathological factors such as lymph node samples, tumor differentiation, vascular or neural invasion, and pathological T stage were examined by two experienced pathologists (Zhongwu Li and Yanhua Bai) based on these H&E slides. As recommended by ESMO guidelines [4, 5], we divided patients into three clinical risk groups. The patients who had one of the following high-risk factors, which included lymph nodes sampling less than 12, poorly differentiated tumors, vascular or neural invasion, pathological T4 stage tumors and clinical presentation with intestinal obstruction or perforation were classified as the high-risk group. Among the rest of patients, those with MSI were believed to have better survival [24] and belonged to the low-risk group, while the remaining patients the general-risk group.

### Immunohistochemistry (IHC) for mismatch repair systems (MMR)

FFPE sections were cut into 4 μm and baked for 1 h at 60 ℃. Then IHC staining was performed automatically by Ventana Benchamrk (Roche, US), an automatic IHC stainer. We used mouse anti-MLH1 monoclonal antibody (Clone ES05, Shanghai GeneTech, Shanghai, China), anti-PMS2 monoclonal antibody (Clone EP51, Shanghai GeneTech, Shanghai, China), anti-MSH2 monoclonal antibody (Clone RED2, Shanghai GeneTech, Shanghai, China) and anti-MSH6 monoclonal antibody (EP49, Shanghai GeneTech, Shanghai, China) to sections of all collected cases to determine MMR, which were recommended in reference and used in our department for several years. The results were evaluated under a microscope by two pathologists independently (Zhongwu Li and Yanhua Bai) unaware of related clinical information. If there was a disagreement between two pathologists, we asked a senior doctor for the final opinion. IHC staining was negative when all tumor cells showed loss of nuclear staining. Tumors were classified as mismatch repair protein-proficient (pMMR), if all IHC staining results were positive, or mismatch repair protein-deficient (dMMR), if at least one protein was negative, which was associated with MSI.

### Tumor sampling

For DNA ploidy, stroma and nucleotyping analyses, we selected one tumor block deemed representative from each patient and annotated the whole epithelial tumor region. The analysis processes were carried out in Ningbo Meishan FTZ MBM Clinical Lab Co., Ltd.

### Measurement of DNA ploidy

Tumor DNA ploidy analysis by image cytometry was performed with DNA Ploidy Working Station (Room4, Kent, UK). Briefly, a 5-μm section, cut and stained with H&E, was used for control and for defining the tumor region. One or two 50-μm sections, containing more than 90% representative tumor tissue, were cut from the FFPE tissue blocks. The sections were used for monolayer preparations and were stained using Feulgen’s method, as previous report [25]. Then an image of each Feulgen-stained nucleus was captured by a high-resolution digital scanner (Aperio AT2, Leica, Germany), and images were automatically grouped into different galleries for tumor nuclei, reference nuclei and discarded nuclei. DNA ploidy histograms were created from the integrated optical density (IOD) of the nuclei using PWS Classifier (Room 4, Kent, UK). Using the reference nuclei as an internal diploid control, DNA ploidy histograms were classified into four groups: diploid, aneuploid, tetraploid and polyploid according to a previous report [25, 26]. In our study, we called aneuploid, tetraploid and polyploid samples collectively as non-diploid.

### Stroma-tumor fraction

The stroma-tumor fraction was automatically calculated by the software Stroma Analyzer (Room 4, Kent, UK). The H&E stained sections were routinely estimated under 10 × 10 lens microscope to select a tumor rich area (with tumor tissue > 50% and necrotic tissue < 10%). Then the images were scanned using an Aperio AT2 digital slide scanner at × 40 (Leica, Germany), giving a resolution of 0.23 µm per pixel. The tumor regions were marked on the scanned images by a highly qualified pathologist using Stroma analyzer (Room 4, Kent, UK) and the stroma fraction was calculated according to the ratio of stroma area to the annotated carcinoma and stroma area automatically. We defined low stroma as a stroma fraction less than or equal to 0.50 and high stroma as a stroma fraction greater than 0.50. The method and cutoff value both referred to a previous study by Danielsen HE and colleagues [21].

### Nuclear texture analysis

Nucleotyping was assessed automatically based on the machine learning algorithm developed by Kleppe and colleagues [20]. Classify each tumor sample by using PWS Classifier and images of tumor nuclei identical to DNA ploidy histograms and quantify chromatin organization by calculating the entropy of pixel grey levels in a subregion of a nucleus. GLEM (grey level entropy matrix), representing the frequency in which each couple of entropy and centre grey level occur throughout a nucleus as well as GLEM4D (a four-dimensional expansion of the GLEM) were produced in Kleppe’s study [20] and an adaptive machine-learning-algorithm was applied to predict the outcome of a patient on the basis of the GLEM4D representation of its tumor [22]. The result was a continuous value termed the chromatin value, which described the total amount of chromatin disorder in a given patient sample [20]. We defined tumors as chromatin homogeneous (CHO) when chromatin values were higher than or equal to 0.044 and chromatin heterogeneous (CHE) when chromatin values less than 0.044 according to Kleppe and colleagues [20].

### Statistic analysis

The endpoints were overall survival (OS) and disease-free survival (DFS). OS was defined as the time from the date of first surgery to the date of death for any reason or the date of the last follow-up. DFS was defined as the time from the date of first surgery to the date of death for any cause or the date of first local recurrence or metastasis. PASS for Windows, Version 15.0 (NCSS Corp, US) was used for calculation of the sample size. IBM SPSS Statistics for Windows, Version 26.0 (IBM Corp, US) software was used for all analyses. Kaplan–Meier survival curves with log-rank tests were plotted to compare OS and DFS. Univariate and multivariate analyses were undertaken using Cox proportional hazard regression models to obtain hazard ratios with 95% confidence interval for parameters. Correlation analyses were performed using Spearman correlation coefficients with a value greater than 0 indicating a positive correlation and a value less than 0 indicating a negative correlation. The statistical significance level was set at 0.05. For each multivariate model established, a two-sided P-value of less than 0.01 was considered statistically significant.

## Results

### Demographic and clinical characteristics

Totally, 417 cases of stage II colon cancer were involved in this study, among which there were 114 cases in the low-risk group, 115 cases in the general-risk group, while 188 cases in the high-risk one. The median age of patients was 61 years, with a male preponderance (63.1% versus 36.9%), a higher proportion of patients (91.4%) who had more than 12 lymph node samplings, and a majority of pT3 patients (84.9% versus pT4 15.1%). In our cohort, a proportion of 38.8% patients were treated with adjuvant chemotherapy (Capecitabine alone, Oxaliplatin, leucovorin and 5-FU, or Oxaliplatin and Capecitabine) while the other 61.2% patients were not after surgery.

The highest proportion of diploid patients was observed in the low-risk group (73.0% versus 43.0% in the general-risk group and 46.8% in the high-risk group). The fraction of patients with a low stroma (low-risk group 76.5% versus general-risk group 86.0% versus high-risk group 86.4%) and patients with chromatin homogeneity, as measured by nucleotyping (low-risk group 97.4% versus general-risk group 80.7% versus high-risk group 81.9%), predominated in all three groups. Other demographic and clinical characteristics of patients were summarized in Table 1.

**Table 1:** Demographic and clinical characteristics of patient

Variables	Total	Low-risk	General-risk	High-risk
	N (%)	N (%)	N (%)	N (%)
Age, years				
≤ 61	212(50.8)	65(56.5)	53(46.5)	94(50.0)
> 61	205(49.2)	50(43.5)	61(53.3)	94(50.0)
Gender				
Male	263(63.1)	73(63.5)	69(60.5)	121(64.4)
Female	154(36.9)	42(36.5)	45(39.5)	67(35.6)
Lymph nodes sampling				
≥ 12	381(91.4)	115(100)	114(100)	152(80.9)
< 12	36(8.6)	/	/	36(19.1)
Mismatch repair status				
pMMR	238(57.1)	/	114(100)	124(66.0)
dMMR	179(42.9)	115(100)	/	64(34.0)
Histological grade				
High	18(4.3)	6(5.2)	5(4.4)	7(3.7)
Middle	334(80.1)	103(89.6)	108(04.7)	123(65.4)
Low	55(13.2)	/	/	55(29.3)
Mucious	10(2.4)	6(5.2)	1(0.9)	3(1.6)
pT stage				
pT3	354(84.9)	115(100)	114(100)	125(66.5)
pT4	63(15.1)	/	/	63(33.5)
Adjuvant chemotherapy				
No	255(61.2)	86(74.8)	87(76.3)	82(43.6)
Yes	162(38.8)	29(25.2)	27(23.7)	106(56.4)
DNA ploidy				
Diploidy	221(53.0)	84(73.0)	49(43.0)	88(46.8)
Non-diploidy	196(47.0)	31(27.0)	65(57.0)	100(53.2)
Stroma				
Low stroma	339(81.3)	88(76.5)	98(86.0)	153(81.4)
High stroma	78(18.7)	27(23.5)	16(14.0)	35(18.6)
Nucleotyping				
Chromatin homogeneous	358(85.9)	112(97.4)	92(80.7)	154(81.9)
Chromatin heterogeneous	59(14.1)	3(2.6)	22(19.3)	34(18.1)
Perforation or obstruction				
No	380(91.1)	115(100)	114(100)	151(80.3)
Yes	37(8.9)	/	/	37(19.7)
Vascular or neurological invasion				
No	362(86.8)	115(100)	114(100)	133(70.7)
Yes	55(13.2)	/	/	55(29.3)

At the end of follow-up, 360 (86.3%) patients were still alive and 52 (12.5%) patients had a recurrence or metastasis. Median OS and median DFS were 92.4 months (25–75% quartiles: 63.5–105.6 months) and 88.2 months (25–75% quartiles: 60.1–104.7 months), respectively.

There was an intermediate positive correlation between DNA ploidy and nucleotyping (coefficient = 0.404, P < 0.001), which meant that non-diploidy and CHE were relevant. A weak positive correlation between pathological T-stage and nucleotyping was observed in our cohort (coefficient = 0.098, P = 0.046), so was between stroma-tumor fraction and nucleotyping (coefficient = 0.141, P = 0.004). Negative correlations were observed between MSI and DNA ploidy (coefficient = − 0.293, P < 0.001) as well as nucleotyping (coefficient = − 0.213, P < 0.001). We did not find correlation between risk group or age and any one of the three parameters of DNA ploidy, stroma and nucleotyping.

### Univariate analyses of prognostic factor

**Fig. 1:**
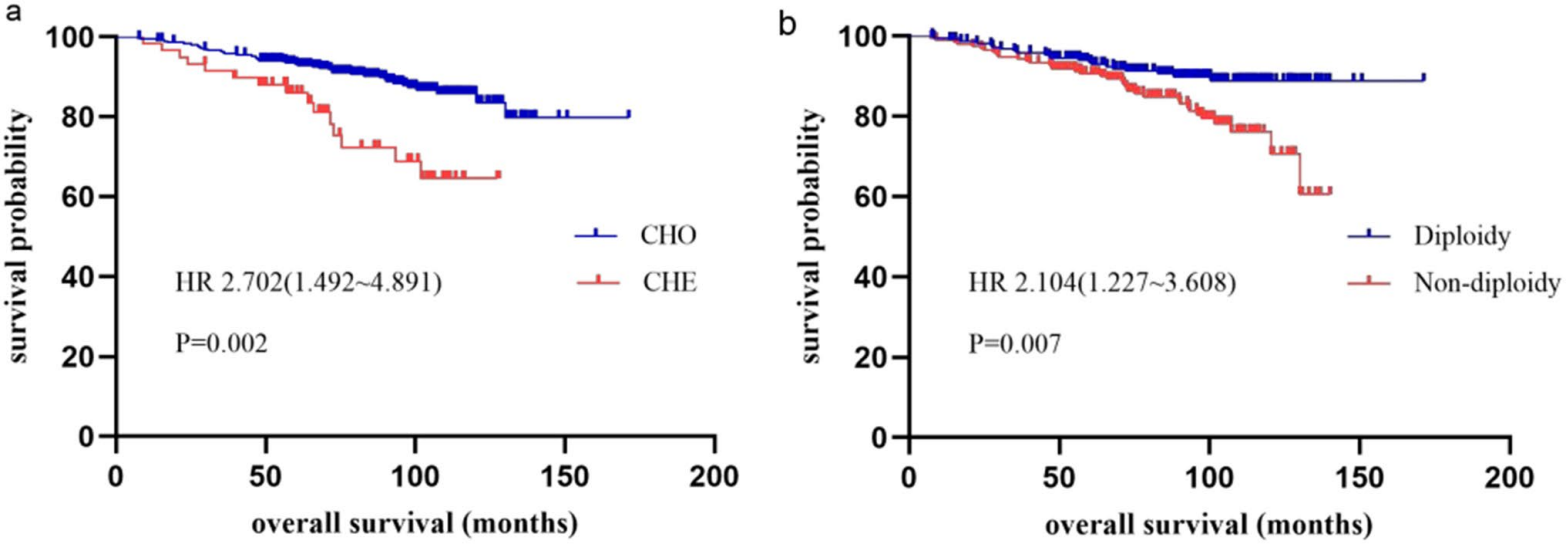
Kaplan–Meier plots illustrating OS for patients that were classified according to nucleotyping (**a**) and DNA ploidy (**b**) in the whole cohort of stage II colon cancer

Our median follow-up time was 91.7 months. We first carried out univariate Cox analysis and the results of OS and DFS in stage II colon cancer were shown in Table 2, results of P value for each group in Supplemental Table S1. Patients in high-risk group had an inferior OS than in non-high-risk group (P = 0.048). For the whole cohort, DNA ploidy and nucleotyping were found to be significant in the analysis of OS (P = 0.007 and P = 0.002, respectively) (Fig. 1). The OS and DFS were both longer in patients of T3 stage than those of T4 (P = 0.005 and P = 0.003, respectively), which was consistent with previous study [27]. Age was the only factor that was significant in OS of all cases as well as in three risk groups (P < 0.001 in the whole cohort, 0.015 in low-risk group, 0.012 in general-risk group, 0.009 in high-risk group, respectively). Significant differences were observed in DNA ploidy of low-risk group (P = 0.010) and nucleotyping of high-risk group (P = 0.009) in terms of OS. In high-risk group, low-stroma patients had a superior DFS than high-stroma patients (P = 0.045), while in other two groups and in all patients, there was no significant statistical difference.

**Table 2:** Univariate analysis on overall survival and disease-free survival of the prognostic factors

Variables	OS		DFS	
	HR(95% CI)	P value	HR(95% CI)	P value
Risk group		0.048		0.113
General and low-risk	1		1	
High-risk	1.719(1.004–2.945)		1.565(0.899–2.722)	
Age, years		< 0.001		0.004
≤ 61	1		1	
> 61	4.666(2.417–9.001)		1.035(1.012–1.060)	
Lymph nodes sampling				0.973
≥ 12	1	0.437	1	
< 12	1.443(0.572–3.639)		0.983(0.353–2.733)	
Mismatch repair status				0.043
pMMR	1	0.203	1	
dMMR	1.420(0.827–2.438)		1.838(1.018–3.318)	
Histological grade		0.923		0.711
High	1		1	
Middle	1.530(0.370–6.319)		1.362(0.330–5.621)	
Low	1.748(0.359–8.508)		0.761(0.139–4.173)	
Mucious	0(0–6.403E + 286)		0(0–3.361E + 298)	
pathological T stage		0.005		0.003
pT3	1		1	
pT4	2.336(1.293–4.218)		2.496(1.369–4.549)	
Adjuvant chemotherapy		0.329		0.336
No	1		1	
Yes	0.753(0.426–1.330)		1.308(0.756–2.263)	
DNA ploidy		0.007		0.089
Diploidy	1		1	
Non-diploidy	2.104(1.227–3.608)		1.612(0.929–2.797)	
Stroma		0.277		0.065
Low stroma	1		1	
High stroma	1.398(0.765–2.556)		1.758(0.965–3.204)	
Nucleotyping		0.002		0.013
Chromatin homogeneous	1		1	
Chromatin heterogeneous	2.702(1.492–4.891)		2.210(1.178–4.144)	
DNA ploidy and stroma		0.021		0.005
Diploidy and low stroma	1		1	
Diploidy and high stroma or non-diploidy and low stroma	1.741(0.960–3.158)		1.076(0.578–2.002)	
Non-diploidy and high stroma	2.956(1.352–6.463)		3.030(1.470–6.244)	
Nucleotyping and stroma		0.016		0.015
Chromatin homogeneous and low stroma	1		1	
Chromatin homogeneous and high stroma or chromatin heterogeneous and low stroma	2.127(1.226–3.691)		1.664(0.907–3.053)	
Chromatin heterogeneous and high stroma	2.303(0.813–6.526)		3.353(1.394–8.064)	
DNA ploidy and nucleotyping		0.001		0.026
Diploidy and chromatin homogeneous	1		1	
Diploidy and chromatin heterogeneous or non-diploidy and chromatin homogeneous	1.638(0.849–3.001)		1.259(0.668–2.372)	
Non-diploidy and chromatin heterogeous	3.539(1.815–6.901)		2.554(1.284–5.079)	
DNA ploidy, stroma, and nucleotyping		0.048		0.025
Diploidy, low stroma, and chromatin homogeneous	1		1	
All other cases	1.870(1.050–3.331)		1.240(0.689–2.230)	
Non-diploidy, high stroma, and chromatin heterogeous	2.949(0.987–8.772)		3.548(1.415–8.899)

According to Danielsen HE and colleagues [21], combining ploidy and stroma stratify stage II tumors patients more accurately, so we combine these three indicators, DNA ploidy, stroma-tumor fraction and nucleotyping, in pairs.

**Fig. 2:**
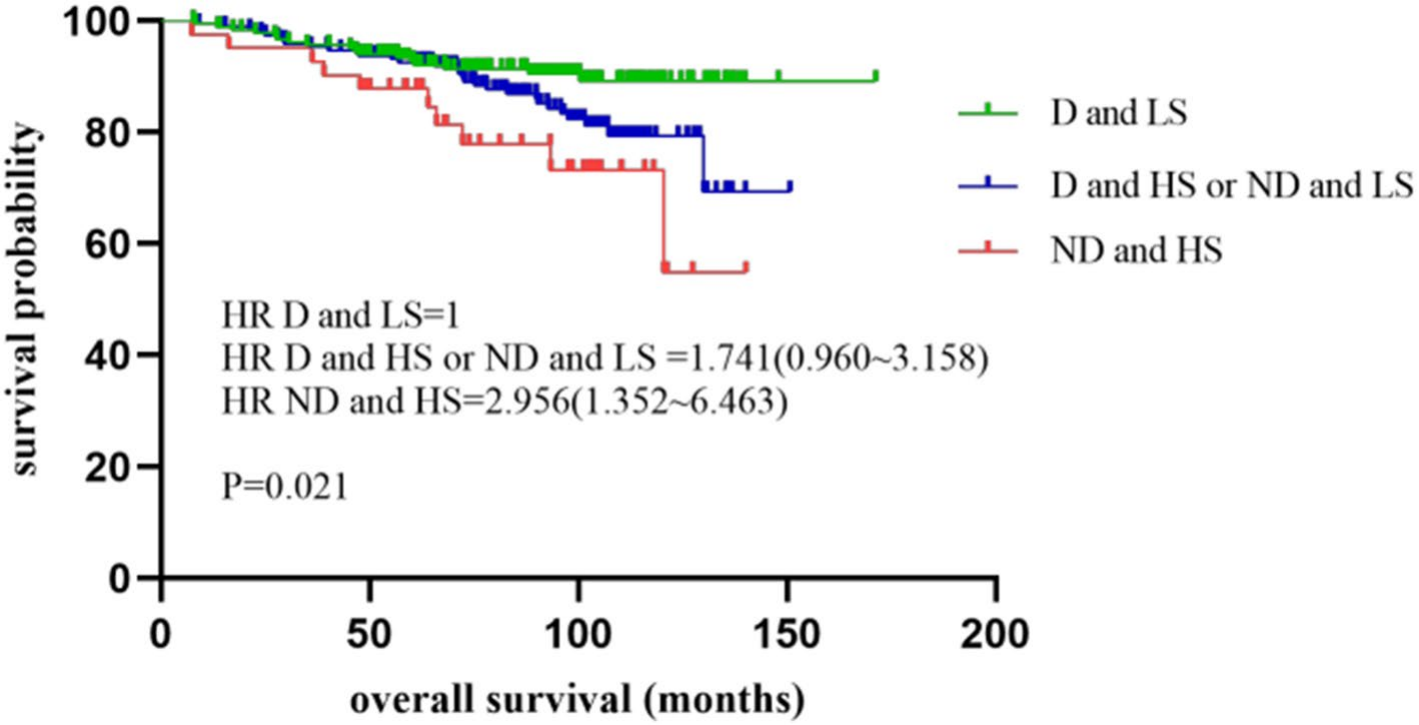
Kaplan–Meier plots illustrating OS for patients that were divided into three subgroups: diploidy and low stroma (D and LS), diploidy and high stroma or non-diploidy and low stroma (D and HS or ND and LS), non-diploidy and high stroma (ND and HS) in the whole cohort of stage II colon cancer

The combination of DNA ploidy and stroma was divided into three subgroups. Patients with low-stroma and diploid had a median OS of 92.9 months (25–75% quartiles: 64.2–110.0 months) and a median DFS of 91.6 months (25–75% quartiles: 60.9–105.0 months), which we defined as low-risk subgroup. Patients with high-stroma and diploid together with low-stroma and non-diploid represented intermediate-risk subgroup, with a median OS of 91.8 months (25–75% quartiles: 64.4–105.9 months) and a median DFS of 88.1 months (25–75% quartiles: 58.0–104.8 months). High-stroma and non-diploid patients who were in high-risk subgroup had a relative lower median OS of 76.4 months (25–75% quartiles: 58.6–104.3 months) and a lower median DFS of 72.3 months (25–75% quartiles: 48.7–102.6 months). The combination of DNA ploidy and stroma was statistically significant for both OS and DFS (P = 0.021 and P = 0.005, respectively) (Table 2 and Fig. 2). As for each risk group, significant differences were observed in analysis of OS (P = 0.002) in low-risk group with a median value of 100.8 months (25–75% quartiles: 78.2–118.5 months) and in analysis of DFS (P = 0.024) in high-risk group with a median value of 66.2 months (25–75% quartiles: 52.5–91.3 months).

**Fig. 3:**
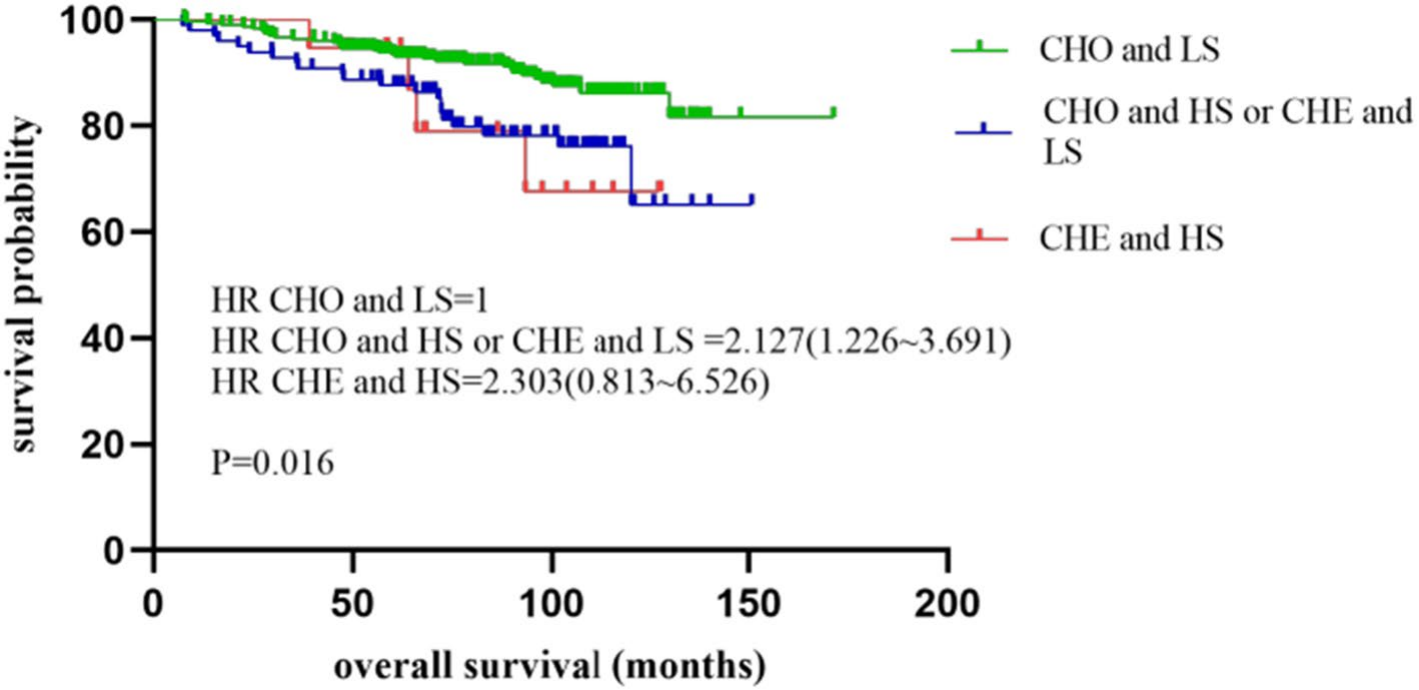
Kaplan–Meier plots illustrating OS for patients that were divided into three subgroups: CHO and low stroma (CHO and LS), CHO and high stroma or CHE and low stroma (CHO and HS or CHE and LS), CHE and high stroma (CHE and HS) in the whole cohort of stage II colon cancer

Similarly, we combined nucleotyping and stroma, which was respectively divided into three subgroups too. The CHO and low stroma subgroup (low-risk subgroup) was found to have the longest median OS of 94.6 months (25–75% quartiles: 64.2–105.2 months) as well as the longest median DFS of 91.7 months (25–75% quartiles: 57.1–105.9 months). While the CHO and high stroma together with CHE and low stroma subgroup had a median OS of 84.5 months (25–75% quartiles: 62.6–108.0 months) and a median DFS of 83.0 months (25–75% quartiles: 57.1–108.0 months), referred to as intermediate-risk subgroup. The CHE and high stroma subgroup (high-risk subgroup) had the shortest median OS of 67.9 months (25–75% quartiles: 58.4–103.8 months) and the shortest median DFS of 67.9 months (25–75% quartiles: 48.5–97.6 months). The combination of nucleotyping and stroma was statistically significant for both OS (P = 0.016) and DFS (P = 0.015) (Table 2 and Fig. 3). When we analyzed three risk groups independently, only in the high-risk group we observed significant differences in OS (P = 0.035) and DFS (P = 0.013).

**Fig. 4:**
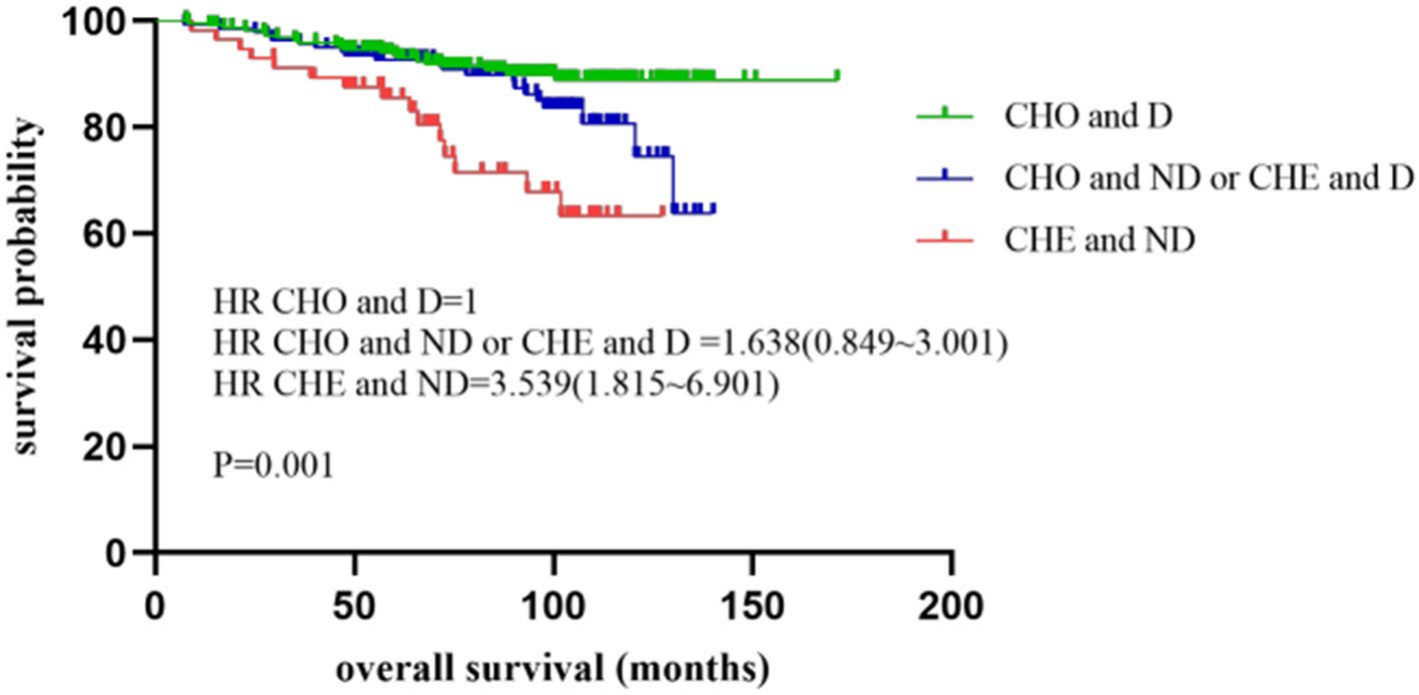
Kaplan–Meier plots illustrating OS for patients that were divided into three subgroups: CHO and diploidy (CHO and D), CHO and non-diploidy or CHE and diploidy (CHO and ND or CHE and D), CHE and non-diploidy (CHE and ND) in the whole cohort of stage II colon cancer

The combination of nucleotyping and DNA ploidy was also tested and divided into three subgroups. The low-risk subgroup (CHO and diploidy) had a median OS of 93.4 months (25–75% quartiles: 68.3–110.8 months) and a median DFS of 91.7 months (25–75% quartiles: 64.2–107.0 months). The intermediate-risk subgroup defined as CHO and non-diploidy together with CHE and diploidy had a median OS of 95.7 months (25–75% quartiles: 63.0–104.9 months) and a median DFS of 90.2 months (25–75% quartiles: 60.9–104.3 months). The high-risk subgroup (CHE and non-ploidy) represented the worst OS with a median value of 69.5 months (25–75% quartiles: 56.6–102.0 months) and DFS with a median value of 67.9 months (25–75% quartiles: 48.7–101.3 months). The results showed statistical significance in both OS (P = 0.001) and DFS (P = 0.026) (Table 2 and Fig. 4). For each risk group, we found significant differences in both low-risk group (P = 0.002 for OS, P = 0.029 for DFS, respectively) and high-risk group (P = 0.008 for OS, P = 0.019 for DFS, respectively).

We further combined DNA ploidy, stroma-tumor fraction and nucleotyping all together. We defined patients with diploidy, low stroma as well as CHO as low-risk subgroup, non-diploidy, high stroma as well as CHE as high-risk subgroup and all other cases were named as intermediate-risk subgroup. We observed statistic significances whether OS or DFS was used as endpoint (P = 0.048 for OS, P = 0.025 for DFS, respectively) for the whole cohort (Table 2). When it came to each risk group, however, no significant differences were observed. And it should be noted that no parameter was significant in general-risk group in our analyses.

### Multivariable analyses of prognostic factor

We performed multivariable analysis with Cox regression. For the whole cohort, DNA ploidy, stroma, nucleotyping, the combination of DNA ploidy and stroma, the combination of nucleotyping and stroma, and the combination of nucleotyping and DNA ploidy were used as independent variables. Age, pathological T stage and mismatch repair status, which was a key prognostic factor [24], were adjusted to establish multivariable models. Nucleotyping [HR 2.412 (95% CI 1.291–4.509) for CHE versus CHO, P = 0.006] and the combination of nucleotyping and DNA ploidy [HR 1.458 (95% CI 0.783–2.718) for intermediate-risk subgroup versus low-risk subgroup, HR 3.042 (95% CI 1.482–6.248) for high-risk subgroup versus low-risk subgroup, P = 0.009] were the dominant contributory factors for OS (Table 3). The three parameters as standalone and combined factors were analyzed in each risk group too (Supplemental Table S2–S4). For low-risk group, we used age as adjusted variable to establish multivariable models and found the combination of nucleotyping and DNA ploidy as the independent prognostic factor [HR 3.415 (95% CI 1.146–10.172) for intermediate-risk subgroup versus low-risk subgroup, HR 55.591 (95% CI 4.594–672.629) for high-risk subgroup versus low-risk subgroup, P = 0.002)] on OS. When we analyzed high-risk group, age and pathological T stage were used as adjusted variables and nucleotyping were found to be the dominant contributory factor [HR 2.999 (95% CI 1.356–6.636) for CHE versus CHO, P = 0.007] for OS. We used age as adjusted variable in general-risk group but observed no significant differences in multivariable Cox models.

**Table 3:** Multivariable analysis of DNA ploidy, stroma, and nucleotyping as standalone or combined factors on overall survival and disease-free survival

Independent variables	OS		DFS	
	HR(95%CI)	P value	HR(95%CI)	P value
DNA ploidy		0.040		0.378
Diploidy	1		1	
Non-diploidy	1.804(1.026–3.173)		1.298(0.727–2.315)	
Stroma		0.176		0.069
Low stroma	1		1	
High stroma	1.525(0.828–2.812)		1.761(0.956–3.242)	
Nucleotyping		0.006		0.092
Chromatin homogeneous	1		1	
Chromatin heterogeneous	2.412(1.291–4.509)		1.753(0.912–3.370)	
DNA ploidy and stroma		0.037		0.015
Diploidy and low stroma	1		1	
Diploidy and high stroma or non-diploidy and low stroma	1.513(0.824–2.780)		0.902(0.478–1.702)	
Non-diploidy and high stroma	2.896(1.287–6.517)		2.495(1.173–5.307)	
Nucleotyping and stroma		0.023		0.064
Chromatin homogeneous and low stroma	1		1	
Chromatin homogeneous and high stroma or chromatin heterogeneous and low stroma	2.044(1.170–3.572)		1.538(0.835–2.836)	
Chromatin heterogeneous and high stroma	2.490(0.853–7.270)		2.770(1.112–6.900)	
DNA ploidy and nucleotyping		0.009		0.198
Diploidy and chromatin homogeneous	1		1	
Diploidy and chromatin heterogeneous or non-diploidy and chromatin homogeneous	1.458(0.783–2.718)		1.076(0.560–2.064)	
Non-diploidy and chromatin heterogeous	3.042(1.482–6.248)		1.885(0.905–3.924)	
DNA ploidy, stroma, and nucleotyping		0.118		0.132
Diploidy, low stroma, and chromatin homogeneous	1		1	
All other cases	1.643(0.910–2.967)		1.053(0.577–1.922)	
Non-diploidy, high stroma, and chromatin heterogeous	2.833(0.908–8.837)		2.569(0.969–6.815)	

All variables were separately adjusted with age, pathological T stage and mismatch repair status; A two-sided P-value of less than 0.01 was considered statistically significant

## Discussion

As recommended by current international guidelines [5], adjuvant chemotherapy was limited to patients with at least one of the high risk factors aforementioned in localized colon cancer. However, there is significant variation in clinical practice among countries, especially in stage II CRC, despite the fact that adjuvant treatment of clinical low risk patients was not associated with improvements in outcomes [28]. Considering the toxic and side effects, high expenses, and many inconveniences including transportation and difficulties in admission to hospital of treatment, as well as the insufficient survival benefit of only an absolute improvement of 3.6% [2], it’s necessary to explore novel biomarkers that can stratify stage II CRC more precisely and can hopefully be accepted by international criteria.

In the present study, we have proven that nucleotyping was the dominant prognostic factor using whether univariate or multivariable Cox analyses in our whole cohorts as well as in high-risk group, as was demonstrated in a previous study [22]. Tumor progression is accompanied by genomic and epigenetic changes, which alter the nuclei in multiple ways including the size of the nucleus, the density of DNA, and the structure of chromatin. Nucleotyping, as a quantitative analysis of the degree of nuclear disorder of tumors, provides descriptions of cell nuclei and in particular the chromatin structure in cancer cell nuclei [29] based on the theory of statistical texture analysis. It can help to distinguish between normal tissue and precancerous tissue [19] and its prognostic significance has been shown in several cancers [30–33]. As for CRC, a pan-cancer study involving stage I and II CRC [20] showed a predictive function towards cancer-specific survival. Our results were similar by finding that patients with CHE had a shorter OS (a median of 69.5 months) than those with CHO (a median of 94.4 months) in all patients. The above evidence confirmed nucleotyping as an independent prognostic factor and could add value to traditional tumor-node-metastasis staging system.

It’s believed that more than 70% solid tumors are aneuploid [7], resulting from chromosome missegregation and correlating with the aggressiveness of the tumor. Though aneuploidy, describing the state and CIN, referring to the elevated rate of chromosome gain or loss, are not synonymous, they are interrelated and aneuploidy can be used representing CIN. Unlike point mutations that affect only a small number of genes, the number alterations of chromosomes alter the transcription of hundreds of genes and can disturb a large array of cellular processes [6]. We detected cellular DNA ploidy as a proxy for the degree of aneuploidy and assumed it to be prognostic in stage II colon cancer as in previous studies of breast, endometrioid endometrial, ovarian and prostate cancers [34]. There was a significant survival advantage in diploidy patients, whose median OS was 87.5 months compared to non-diploidy patients, whose median OS was 93.4 months. But in multivariable analysis, significance was not observed. Considering the result that there was a negative correlation between DNA ploidy and MSI and the view that CIN was strongly negatively associated with MSI in a previous analysis of VICTOR trial [10], the difference might be offset in multivariable models. We also observed a superior survival in diploidy patients of low-risk group with dMMR, and the consequence was consistent with the study of VICTOR trail [10] too. The unobserved significance in multivariable analysis was possibly attributed to our small sample of low-risk patients.

It was identified that CHE patients were more likely to have non-diploid phenotypes, and diploid patients were also more likely to be CHO by previous researchers [23], suggesting that both biomarkers correlated on a cellular level and the correlation was demonstrated in our data (with a coefficient of 0.404). It was reasonable to observe significance when we carried out analysis of the combination of DNA ploidy and nucleotyping based on the above results. Patients with CHE and non-ploidy had the worst prognosis in the whole cohorts and in the low-risk group as well. The large-scale genomic instability owing to aneuploidy must correlate with large-scale rearrangement of interphase nuclear chromatin, which can be reflected by examining nucleotyping. Thus the correlation could be explained properly. After verification by multivariable models, the combination of DNA ploidy and nucleotyping was proven to be an independent predictor of survival in stage II colon cancer and a candidate marker to select those with higher risk even in low-risk clinical group with MSI.

Stroma surrounding tumors was associated with tumor initiation, progression, and metastasis and held prognostic value [15]. Researchers suggested that stroma supplied the tumor with growth factors, cytokines, and metabolites, and stimulated blood vessel formation, which could cause tumorigenesis and induction of epithelial-mesenchymal transition (EMT) [35]. And an expanded tumor stroma might influence disease progression by promoting tumor growth and enhancing invasive capabilities. Vascular and lymphatic metastases were more likely with the increasing of stroma ratio [36]. Contradictory to the results earlier that high stroma was associated with poorer survival whether in domestic populations [17] or in European populations [21, 37], we found no significance between low stroma and high stroma whether using OS or DFS as an endpoint despite a tendency in DFS. The possible causes might include the variations in methods when calculating the stroma-tumor fraction, as we evaluated all tumor areas on the whole scanned images rather than studied the areas with the deepest tumor infiltrating margin, and the small sample size, as well as the correlated low power calculation. Given the characteristics of peritumoral stroma in angiogenesis, the stroma-tumor fraction, or tumor stroma percentage according to Park JH and colleagues [36], was a potential biomarker to select patients who could be considered for treatment targeted at the stroma itself and beneficial from anti-angiogenic therapies.

Our results were consistent with what Danielsen HE and colleagues [21] had suggested that the combination of DNA ploidy and stroma-tumor fraction provided a prognostic stratifier for stage II CRC even superior to RNA signature. Similarly, we found that this combined indicator was statistically significant based on whether OS or DFS, displaying predictive functions in survival as well as in recurrence and metastasis in colon patients. An assumption was that the effect of aneuploidy may not be driven by a particular combination of chromosomes per se, but rather by the specific interaction of the karyotype with the various genetic contexts and microenvironments found in different tissues [6]. Although mechanism not elucidated, tumor stroma as the most important microenvironment could have an effect on ploidy phenotypes and evolving directions [38]. Thus a deeper understanding of the relationship between ploidy and stroma might reveal new avenues for anti-cancer therapies. Though we did not found significant statistical difference between low-stroma and high-stroma patients except in high-risk group, the combination of DNA ploidy and stroma as well as the combination of nucleotyping and stroma turned out to have prognostic value. This might be due to the significance of DNA ploidy and nucleotyping take dominant position when we combine the two factors with stroma.

Our current study emphasized the important prognostic roles of conventional risk factors including age and pathological T stage, and added new biomarkers that were valuable to decision-making in postoperative adjuvant treatment. MSI was the most validated prognostic marker next to clinical prognostic factors, which has been demonstrated by a meta-analysis [39], and showed statistical significance only in DFS in the whole cohort (P = 0.043), suggesting a decreased rate of events including relapse and metastasis. Our bias when selecting cases in different risk groups may affect the accuracy. While previous studies have shown that in multivariable analysis, the absence of adjuvant chemotherapy was risk factor for stage II colon cancer [37], our study showed that adjuvant chemotherapy had no significant effect on prognosis whether in all patients or in each risk group. The inferred reasons were as following: variations between countries and ethnic groups; the limitations of diagnostic and surgical level twenty years ago; the younger median age in our cohort than that of study mentioned above (with a median age of 73 years old); the refusal to chemotherapy in our cohorts due to financial reason or utilization of Chinese medicine as a substitute.

According to Chinese Protocol of Diagnosis and Treatment of Colorectal Cancer (2023 Edition), adjuvant therapy is needed only in high-risk group with a recommended chemotherapy regimen of CapeOx or FOLFOX based on Oxaliplatin, or monotherapy with 5-FU/LV and Capecitabine. However, using the two independent prognostic factors produced in our study, we tried adjusting treatment strategies, considering if patients with diploidy and CHO could avoid adjuvant treatment even in high-risk group. Postoperative adjuvant chemotherapy was not recommended in CRC with dMMR or MSI-H. A possible treatment change may be that patients with non-diploidy and CHE should accept at least monotherapy, though they belonged to low-risk group. On the basis of these results, we can infer that stratification of patients according to nucleotyping and the combination of ploidy and nucleotyping will help select stage II colon patients who may benefit from postoperative adjuvant therapy, avoid overtreatment and insufficient clinical intervention. By this way, the novel biomarkers may help guide clinical decisions, optimize prognosis assessments, and tailor individual treatments.

In patients who were older than 61 years old, CHE and the combination of non-ploidy and CHE represented shorter OS time (P = 0.009 for nucleotyping and P = 0.016 for the combination of ploidy and nucleotyping). While in patients who were at pT4 stage, CHE and the combination of non-ploidy and CHE were associated with inferior DFS (P = 0.013 for nucleotyping and P = 0.034 for the combination of ploidy and nucleotyping). It was possible to take the novel parameters and existing conventional indicators such as age and pathological T stage into comprehensive consideration in clinical practice to develop the best diagnosis and treatment plan.

One of the obvious restrictions is the single-centre design with a relatively small sample size. Though we tried to collect patients who met our inclusion criteria regardless of gender or age, limited cases were included. The reasons were that electronic information might lost due to time factor and that there were a lower early cancer diagnosis rate and a lower proportion of stage II colon cancer patients in China due to the limited coverage of cancer screening. Other restrictions included the retrospective character, inconsistence of follow-up time, variations in adjuvant chemotherapy regiments, different compliance of regular examinations, and an inherent disadvantage of OS susceptible to non-cancer-related deaths. However, we still successfully identified two independent prognostic indicators—nucleotyping and the combination of nucleotyping and DNA ploidy, and might stratify stage II colon cancers more accurately, therefore reducing adjuvant overtreatment and intensifying adjuvant treatment on those at a higher risk of death [20]. We expanded the application of nucleotyping as a predictor from high risk stage II colon cancer [22] to whole risks. Given the speed, simplicity, accuracy, batch processing, and low cost of nucleotyping measurements [19], it will become more competitive in the clinical environment and contribute to personalized medication in oncology.

## Conclusion

Nucleotyping as well as the combination of DNA ploidy and nucleotyping were confirmed as independent prognostic predictors in stage II colon cancer patients of all risks, and they were hopeful novel biomarkers that might contribute in readjusting traditional clinical grouping. Nucleotyping, with some explicit advantages, may help in clinical decision-making and individualized therapy strategies in the future.

### Supplementary Information

Additional file 1.

## Data Availability

All data not included in the manuscript are available from the corresponding author upon reasonable request.
