# Supplementary material for: Exploring prognostic values of DNA ploidy, stroma-tumor fraction and nucleotyping in stage II colon cancer patients
Source: Discov Oncol. 2024 Jun 14;15:227. doi: 10.1007/s12672-024-01087-w (PMC11178745; doi:10.1007/s12672-024-01087-w)
Supplement: Supplementary file 1 — Additional file 1. [file 12672_2024_1087_MOESM1_ESM.docx]

**Supplemental Tables**

| **Supplemental Table 1 The P values in univariate analysis on overall survival and disease-free survival for each group** | | | | | | |
| --- | --- | --- | --- | --- | --- | --- |
| Variables | low-risk | | general-risk | | high-risk | |
|  | OS | DFS | OS | DFS | OS | DFS |
| Age, years | 0.015 | 0.148 | 0.012 | 0.206 | 0.009 | 0.143 |
| Lymph nodes sampling | / | / | / | / | 0.950 | 0.571 |
| Mismatch repair status | / | / | / | / | 0.248 | 0.243 |
| Histological grade | 0.672 | 0.789 | 0.796 | 0.783 | 0.823 | 0.295 |
| pathological T stage | / | / | / | / | 0.058 | 0.016 |
| Adjuvant chemotherapy | 0.690 | 0.517 | 0.128 | 0.947 | 0.154 | 0.757 |
| DNA ploidy | 0.010 | 0.200 | 0.851 | 0.218 | 0.059 | 0.066 |
| Stroma | 0.634 | 0.907 | 0.578 | 0.164 | 0.348 | 0.045 |
| Nucleotyping | 0.114 | 0.086 | 0.540 | 0.470 | 0.003 | 0.008 |
| DNA ploidy and stroma | 0.046 | 0.689 | 0.797 | 0.128 | 0.151 | 0.024 |
| Nucleotyping and stroma | 0.521 | 0.801 | 0.686 | 0.313 | 0.035 | 0.013 |
| DNA ploidy and nucleotyping | 0.002 | 0.029 | 0.741 | 0.457 | 0.008 | 0.019 |
| DNA ploidy, stroma, and nucleotyping | 0.112 | 0.463 | 0.899 | 0.173 | 0.191 | 0.085 |

| **Supplemental Table 2 Multivariable analysis of DNA ploidy, stroma, and nucleotyping as standalone or combined factors on overall survival and disease-free survival in low-risk group** | | | | |
| --- | --- | --- | --- | --- |
| Independent variables | OS | | DFS | |
|  | HR(95% CI) | P value | HR(95% CI) | P value |
| DNA ploidy |  | 0.013 |  | 0.206 |
| Diploidy | 1 |  | 1 |  |
| Non-diploidy | 3.845(1.333~11.097) |  | 2.336(0.627~8.711) |  |
| Stroma |  | 0.350 |  | 0.920 |
| Low stroma | 1 |  | 1 |  |
| High stroma | 1.763(0.537~5.789) |  | 1.085(0.221~5.318) |  |
| Nucleotyping |  | 0.027 |  | 0.034 |
| Chromatin homogeneous | 1 |  | 1 |  |
| Chromatin heterogeneous | 12.205(1.328~112.187) |  | 11.990(1.214~118.453) |  |
| DNA ploidy and stroma |  | 0.016 |  | 0.584 |
| Diploidy and low stromoa | 1 |  | 1 |  |
| Diploidy and high stroma or non-diploidy and low stroma | 2.045(0.621~6.739) |  | 1.624(0.404~6.526) |  |
| Non-diploidy and high stroma | 8.501(1.973~36.633) |  | 2.944(0.324~26.761) |  |
| Nucleotyping and stroma |  | 0.285 |  | 0.662 |
| Chromatin homogeneous and low stroma | 1 |  | 1 |  |
| Chromatin homogeneous and high stroma or chromatin heterogeneous and low stroma | 2.489(0.806~7.684) |  | 1.921(0.470~7.857) |  |
| Chromatin heterogeneous and high stroma | 0.000(0~) |  | 0.000(0~) |  |
| DNA ploidy and nucleotyping |  | 0.002 |  | 0.015 |
| Diploidy and chromatin homogeneous | 1 |  | 1 |  |
| Diploidy and chromatin heterogeneous or non-diploidy and chromatin homogeneous | 3.415(1.146~10.172) |  | 1.793(0.428~7.511) |  |
| Non-diploidy and chromatin heterogeous | 55.591(4.594~672.629) |  | 33.039(3.105~351.523) |  |
| DNA ploidy, stroma, and nucleotyping |  | 0.076 |  | 0.392 |
| Diploidy, low stroma, and chromatin homogeneous | 1 |  | 1 |  |
| all other cases | 2.710(0.902~8.141) |  | 1.781(0.475~6.676) |  |
| Non-diploidy, high stroma, and chromatin heterogeous | / |  | / |  |

All variables were separately adjusted with age; A two-sided P-value of less than 0.01 was considered statistically significant.

| **Supplemental Table 3 Multivariable analysis of DNA ploidy, stroma, and nucleotyping as standalone or combined factors on overall survival and disease-free survival in general-risk group** | | | | |
| --- | --- | --- | --- | --- |
| Independent variables | OS | | DFS | |
|  | HR(95%CI) | P value | HR(95%CI) | P value |
| DNA ploidy |  | 0.899 |  | 0.215 |
| Diploidy | 1 |  | 1 |  |
| Non-diploidy | 0.938(0.349~2.520) |  | 0.535(0.199~1.438) |  |
| Stroma |  | 0.533 |  | 0.154 |
| Low stroma | 1 |  | 1 |  |
| High stroma | 1.492(0.424~5.246) |  | 2.284 (0.733~7.111) |  |
| Nucleotyping |  | 0.550 |  | 0.483 |
| Chromatin homogeneous | 1 |  | 1 |  |
| Chromatin heterogeneous | 1.412(0.455~4.381) |  | 0.588(0.134~2.591) |  |
| DNA ploidy and stroma |  | 0.902 |  | 0.098 |
| Diploidy and low stromoa | 1 |  | 1 |  |
| Diploidy and high stroma or non-diploidy and low stroma | 1.265(0.432~3.704) |  | 0.394(0.128~1.207) |  |
| Non-diploidy and high stroma | 1.002(0.117~8.591) |  | 1.738(0.458~6.585) |  |
| Nucleotyping and stroma |  | 0.665 |  | 0.307 |
| Chromatin homogeneous and low stroma | 1 |  | 1 |  |
| Chromatin homogeneous and high stroma or chromatin heterogeneous and low stroma | 1.623(0.554~4.756) |  | 0.539(0.120~2.410) |  |
| Chromatin heterogeneous and high stroma | 1.432(0.183~11.213) |  | 2.462(0.549~11.644) |  |
| DNA ploidy and nucleotyping |  | 0.769 |  | 0.455 |
| Diploidy and chromatin homogeneous | 1 |  | 1 |  |
| Diploidy and chromatin heterogeneous or non-diploidy and chromatin homogeneous | 0.778(0.247~4.313) |  | 0.570(0.191~1.703) |  |
| Non-diploidy and chromatin heterogeous | 1.262(0.369~6.248) |  | 0.464(0.100~2.148) |  |
| DNA ploidy, stroma, and nucleotyping |  | 0.916 |  | 0.157 |
| Diploidy, low stroma, and chromatin homogeneous | 1 |  | 1 |  |
| All other cases | 1.219 (0.416~3.570) |  | 0.452(0.156~1.304) |  |
| Non-diploidy, high stroma, and chromatin heterogeous | 1.420(0.166~12.183) |  | 1.820(0.385~8.602) |  |

All variables were separately adjusted with age; A two-sided P-value of less than 0.01 was considered statistically significant.

| **Supplemental Table 4 Multivariable analysis of DNA ploidy, stroma, and nucleotyping as standalone or combined factors on overall survival and disease-free survival in high-risk group** | | | | |
| --- | --- | --- | --- | --- |
| Independent variables | OS | | DFS | |
|  | HR(95%CI) | P value | HR(95%CI) | P value |
| DNA ploidy |  | 0.116 |  | 0.088 |
| Diploidy | 1 |  | 1 |  |
| Non-diploidy | 1.948(0.849~4.471) |  | 2.059(0.899~4.718) |  |
| Stroma |  | 0.380 |  | 0.087 |
| Low stroma | 1 |  | 1 |  |
| High stroma | 1.482(0.616~3.567) |  | 2.039 (0.733~7.111) |  |
| Nucleotyping |  | 0.007 |  | 0.016 |
| Chromatin homogeneous | 1 |  | 1 |  |
| Chromatin heterogeneous | 2.999(1.356~6.636) |  | 2.625(0.901~4.614) |  |
| DNA ploidy and stroma |  | 0.197 |  | 0.031 |
| Diploidy and low stromoa | 1 |  | 1 |  |
| Diploidy and high stroma or non-diploidy and low stroma | 1.495(0.599~3.727) |  | 1.368(0.536~3.493) |  |
| Non-diploidy and high stroma | 2.742(0.911~8.249) |  | 3.604(1.296~10.017) |  |
| Nucleotyping and stroma |  | 0.059 |  | 0.030 |
| Chromatin homogeneous and low stroma | 1 |  | 1 |  |
| Chromatin homogeneous and high stroma or chromatin heterogeneous and low stroma | 2.270(1.008~5.114) |  | 2.431(1.064~5.555) |  |
| Chromatin heterogeneous and high stroma | 3.343(0.923~12.114) |  | 3.739(1.184~11.808) |  |
| DNA ploidy and nucleotyping |  | 0.018 |  | 0.038 |
| Diploidy and chromatin homogeneous | 1 |  | 1 |  |
| Diploidy and chromatin heterogeneous or non-diploidy and chromatin homogeneous | 1.298(0.498~3.384) |  | 1.464(0.563~3.805) |  |
| Non-diploidy and chromatin heterogeous | 3.536(1.381~9.053) |  | 3.262(1.279~8.322) |  |
| DNA ploidy, stroma, and nucleotyping |  | 0.222 |  | 0.128 |
| Diploidy, low stroma, and chromatin homogeneous | 1 |  | 1 |  |
| All other cases | 1.588 (0.653~3.861) |  | 1.618 (0.662~3.954) |  |
| Non-diploidy, high stroma, and chromatin heterogeous | 3.328 (0.839~13.197) |  | 3.627(1.044~12.597) |  |

All variables were separately adjusted with age and pathological T stage; A two-sided P-value of less than 0.01 was considered statistically significant.
